# [Bis[μ-bis­(diphenyl­phosphino)methane-1:2κ^2^ *P*:*P*′]-bis­(nitrito-κ^2^ *O*,*O*′)]disilver(I) acetonitrile disolvate

**DOI:** 10.1107/S1600536812041931

**Published:** 2012-10-13

**Authors:** Xue Yang, Xu Huang, Qi-Ming Qiu, Qiong-Hua Jin, Cun-Lin Zhang

**Affiliations:** a Department of Chemistry, Capital Normal University, Beijing 100048, People’s Republic of China; b Key Laboratory of Terahertz Optoelectronics, Ministry of Education, Department of Physics, Capital Normal University, Beijing 100048, People’s Republic of China

## Abstract

The title complex, [Ag_2_(NO_2_)_2_(C_25_H_22_P_2_)_2_]·2CH_3_CN, is a centrosymmetric dimer in which two bis(diphenylphosphino)methane ligands bridge two Ag^+^ ions, forming an eight-membered ring with a short Ag⋯Ag separation of 3.1809 (5) Å. The distorted P_2_O_2_ coordination of the cation is completed by two O-donors from a symmetric bidentate chelate NO_2_^−^ anion [Ag—O = 2.550 (3) and 2.567 (3) Å].

## Related literature

The coordination chemistry of silver(I) complexes has been extensively studied, see: Bowmaker *et al.* (1993 ▶); Cui, Hu *et al.* (2010 ▶); Cui, Jin *et al.* (2010 ▶); Jin, Hu *et al.* (2010 ▶); Jin, Song *et al.* (2010 ▶); Meijboom *et al.* (2009 ▶). For related structures, see: Effendy *et al.* (2004 ▶); Jin *et al.* (2008 ▶); Ma *et al.* (2009 ▶); Song *et al.* (2010 ▶).
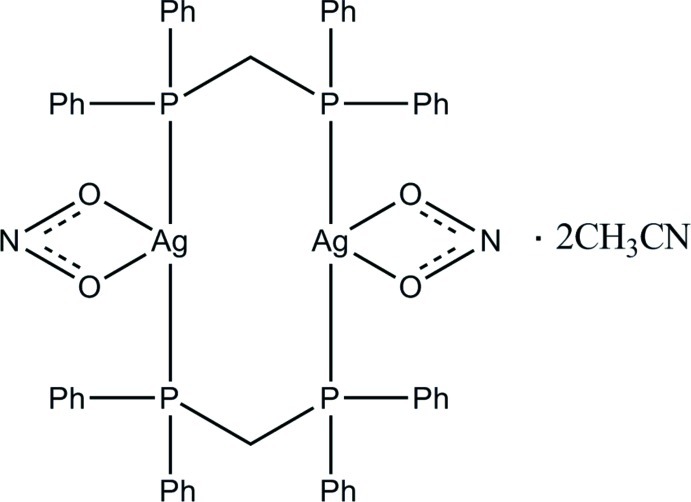


## Experimental

### 

#### Crystal data

[Ag_2_(NO_2_)_2_(C_25_H_22_P_2_)_2_]·2C_2_H_3_N
*M* *_r_* = 1158.60
Monoclinic, 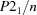

*a* = 12.1390 (11) Å
*b* = 11.1247 (9) Å
*c* = 20.0350 (18) Å
β = 95.543 (1)°
*V* = 2692.9 (4) Å^3^
*Z* = 2
Mo *K*α radiation
μ = 0.89 mm^−1^
*T* = 298 K
0.40 × 0.35 × 0.33 mm

#### Data collection

Bruker SMART CCD area detector diffractometer
Absorption correction: multi-scan (*SADABS*; Bruker, 2007 ▶) *T*_min_ = 0.717, *T*_max_ = 0.757
13205 measured reflections
4752 independent reflections
3845 reflections with *I* > 2σ(*I*)
*R*_int_ = 0.035

#### Refinement

*R*[*F* ^2^ > 2σ(*F* ^2^)] = 0.032
*wR*(*F* ^2^) = 0.090
*S* = 1.07
4752 reflections
308 parameters
3 restraints
H-atom parameters constrained
Δρ_max_ = 1.01 e Å^−3^
Δρ_min_ = −0.46 e Å^−3^

### 

Data collection: *SMART* (Bruker, 2007 ▶); cell refinement: *SAINT-Plus* (Bruker, 2007 ▶); data reduction: *SAINT-Plus*; program(s) used to solve structure: *SHELXS97* (Sheldrick, 2008 ▶); program(s) used to refine structure: *SHELXL97* (Sheldrick, 2008 ▶); molecular graphics: *SHELXTL* (Sheldrick, 2008 ▶); software used to prepare material for publication: *SHELXTL*.

## Supplementary Material

Crystal structure: contains datablock(s) global, I. DOI: 10.1107/S1600536812041931/zs2232sup1.cif

Structure factors: contains datablock(s) I. DOI: 10.1107/S1600536812041931/zs2232Isup2.hkl

Additional supplementary materials:  crystallographic information; 3D view; checkCIF report

## Figures and Tables

**Fig. 1.**
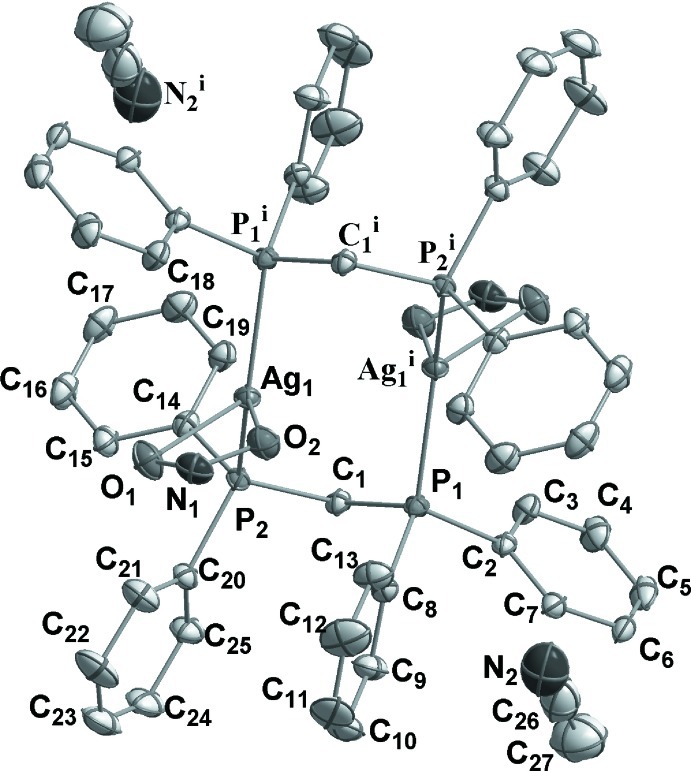
The molecular [Ag2(C25H22P2)2(NO2)2].2(CH3CN), showing the atom-numbering scheme, with displacement ellipsoids drawn at the 30% probability level. For symmetry code (i): -x+1, -y+1, -z+1.

## supplementary crystallographic information

### Comment

The coordination chemistry of silver(I) complexes has been extensively studied because of their luminescence properties and potential applications in catalysis, photography, antimicrobial activities and electrochemical processes (Bowmaker *et al.*, 1993; Cui, Hu *et al.*, 2010; Cui, Jin *et al.*, 2010; Jin, Hu *et al.*, 2010; Jin, Song *et al.*, 2010; Meijboom *et al.*, 2009). Recently, some silver(I) complexes containing phosphine ligands and coordinated anions have been reported (Jin *et al.*, 2008; Song *et al.*, 2010). Continuing these efforts, we synthesized a new complex using AgNO_2_, bis(diphenylphosphino)methane (dppm) and 1,2-bis(4-pyridyl)ethane, the title compound [Ag_2_(dppm)_2_(NO_2_)_2_] . 2(CH_3_CN), and the structure is reported here.

In the title compound two silver atoms are bridged by two dppm ligands, giving a centrosymmetric dimer (Fig. 1) having a Ag···Ag distance of 3.1809 (5) Å, which is longer than that found in the analogous complex [Ag_2_*L*_2_](SO_3_CF_3_)_2_ (*L* = 4'-phenylterpyridine) [2.9452 (4) Å] (Ma *et al.*, 2009). Each of the two NO_2_^-^ anions chelates an Ag atom with Ag—O bond distances of 2.550 (3) and 2.567 (3) Å, so each Ag atom is tetracoordinated. These values compare with 2.694 (4) and 2.559 (5) Å in a similar crystal [AgNO_2_(dppm)]_2_, which was synthesized in methanol (Effendy *et al.*, 2004). In the title complex, the P2—Ag1—P1 angle is 144.82 (3)°, the O1—Ag1—O2 angle is 47.98 (10)°, while the P—Ag1—O angles are in the range of 85.18 (7)–129.41 (7)°, indicating a very distorted tetrahedral stereochemistry about the two silver(I) atoms. The Ag1—P1 and Ag1—P2 bond lengths are 2.4747 (8) and 2.4395 (9) Å, which are both longer than the Ag—P bond found in [Ag(NCS)(C_25_H_22_P_2_)]_n_ (Song *et al.*, 2010), which is analogous to the title compound, synthesized using a similar reaction in the presence of 1,10-phenanthroline.

### Experimental

The title complex was synthesized using the following procedure. Bis(diphenylphosphino)methane (dppm, 0.0769 g, 0.2 mmol) was added to a stirred solution of AgNO_2_ (0.0308 g, 0.2 mmol) and 1,2-bis(4-pyridyl)ethane (0.0368 g, 0.2 mmol) in a mixture of CH_3_CN (5 ml) and H_2_O (5 ml). Stirring was continued for 6 h at room temperature, after which the white precipitate was filtered off. Subsequent slow evaporation of the colorless filtrate at ambient temperature resulted in the formation of colorless crystals of the title complex. Crystals suitable for single-crystal X-ray diffraction were selected directly from the sample as prepared.

### Refinement

The final refinements were performed with isotropic thermal parameters. All hydrogen atoms were located in calculated sites with C—H = 0.93 Å (aromatic), 0.96 Å (methyl) or 0.97 Å (methylene) and included in the refinement in the riding model approximation with *U*_iso_(H) = 1.2*U*_eq_(aromatic and methylene C) or 1.5*U*_eq_(methyl C).

### Crystal data

**Table:**

[Ag_2_(NO_2_)_2_(C_25_H_22_P_2_)_2_]·2C_2_H_3_N	*F*(000) = 1176
*M**_r_* = 1158.60	*D*_x_ = 1.429 Mg m^−^^3^
Monoclinic, *P*2_1_/*n*	Mo *K*α radiation, λ = 0.71073 Å
Hall symbol: -P 2yn	Cell parameters from 6670 reflections
*a* = 12.1390 (11) Å	θ = 2.5–28.2°
*b* = 11.1247 (9) Å	µ = 0.89 mm^−^^1^
*c* = 20.0350 (18) Å	*T* = 298 K
β = 95.543 (1)°	Block, colourless
*V* = 2692.9 (4) Å^3^	0.40 × 0.35 × 0.33 mm
*Z* = 2	

### Data collection

**Table:**

Bruker SMART CCD area detector diffractometer	4752 independent reflections
Radiation source: fine-focus sealed tube	3845 reflections with *I* > 2σ(*I*)
Graphite monochromator	*R*_int_ = 0.035
φ and ω scans	θ_max_ = 25.0°, θ_min_ = 2.5°
Absorption correction: multi-scan (*SADABS*; Bruker, 2007)	*h* = −14→13
*T*_min_ = 0.717, *T*_max_ = 0.757	*k* = −13→13
13205 measured reflections	*l* = −23→18

### Refinement

**Table:**

Refinement on *F*^2^	Primary atom site location: structure-invariant direct methods
Least-squares matrix: full	Secondary atom site location: difference Fourier map
*R*[*F*^2^ > 2σ(*F*^2^)] = 0.032	Hydrogen site location: inferred from neighbouring sites
*wR*(*F*^2^) = 0.090	H-atom parameters constrained
*S* = 1.07	*w* = 1/[σ^2^(*F*_o_^2^) + (0.037*P*)^2^ + 2.4337*P*] where *P* = (*F*_o_^2^ + 2*F*_c_^2^)/3
4752 reflections	(Δ/σ)_max_ = 0.002
308 parameters	Δρ_max_ = 1.01 e Å^−^^3^
3 restraints	Δρ_min_ = −0.46 e Å^−^^3^

### Special details

**Table:**

Geometry. All e.s.d.'s (except the e.s.d. in the dihedral angle between two l.s. planes) are estimated using the full covariance matrix. The cell e.s.d.'s are taken into account individually in the estimation of e.s.d.'s in distances, angles and torsion angles; correlations between e.s.d.'s in cell parameters are only used when they are defined by crystal symmetry. An approximate (isotropic) treatment of cell e.s.d.'s is used for estimating e.s.d.'s involving l.s. planes.
Refinement. Refinement of *F*^2^ against ALL reflections. The weighted *R*-factor *wR* and goodness of fit *S* are based on *F*^2^, conventional *R*-factors *R* are based on *F*, with *F* set to zero for negative *F*^2^. The threshold expression of *F*^2^ > σ(*F*^2^) is used only for calculating *R*-factors(gt) *etc*. and is not relevant to the choice of reflections for refinement. *R*-factors based on *F*^2^ are statistically about twice as large as those based on *F*, and *R*- factors based on ALL data will be even larger.

### Fractional atomic coordinates and isotropic or equivalent isotropic displacement parameters (Å^2^)

**Table:**

	*x*	*y*	*z*	*U*_iso_*/*U*_eq_	
Ag1	0.43541 (2)	0.61579 (2)	0.521730 (12)	0.03586 (10)	
P1	0.55178 (7)	0.70130 (8)	0.43866 (4)	0.0324 (2)	
P2	0.43562 (7)	0.53526 (8)	0.63500 (4)	0.0327 (2)	
N1	0.2195 (3)	0.6845 (4)	0.44450 (19)	0.0626 (10)	
N2	0.9358 (7)	0.8704 (8)	0.6794 (5)	0.154 (3)	
O1	0.2546 (3)	0.7319 (3)	0.49890 (16)	0.0729 (9)	
O2	0.2823 (2)	0.6093 (3)	0.42403 (16)	0.0627 (7)	
C1	0.5182 (3)	0.6221 (3)	0.35872 (17)	0.0369 (8)	
H1A	0.5540	0.6623	0.3236	0.044*	
H1B	0.4389	0.6247	0.3467	0.044*	
C2	0.5182 (3)	0.8565 (3)	0.41655 (17)	0.0373 (8)	
C3	0.4172 (3)	0.8834 (3)	0.3812 (2)	0.0498 (10)	
H3	0.3691	0.8215	0.3669	0.060*	
C4	0.3871 (4)	1.0011 (4)	0.3668 (2)	0.0649 (12)	
H4	0.3198	1.0177	0.3424	0.078*	
C5	0.4561 (5)	1.0931 (4)	0.3883 (2)	0.0682 (14)	
H5	0.4352	1.1722	0.3791	0.082*	
C6	0.5555 (4)	1.0693 (4)	0.4234 (2)	0.0619 (12)	
H6	0.6021	1.1322	0.4381	0.074*	
C7	0.5875 (3)	0.9515 (3)	0.43718 (19)	0.0485 (9)	
H7	0.6561	0.9359	0.4605	0.058*	
C8	0.7019 (3)	0.6971 (3)	0.45118 (18)	0.0412 (8)	
C9	0.7677 (3)	0.7389 (4)	0.4033 (2)	0.0649 (12)	
H9	0.7354	0.7722	0.3635	0.078*	
C10	0.8810 (4)	0.7309 (6)	0.4150 (3)	0.0925 (18)	
H10	0.9250	0.7602	0.3831	0.111*	
C11	0.9295 (4)	0.6809 (6)	0.4720 (4)	0.110 (2)	
H11	1.0063	0.6755	0.4788	0.131*	
C12	0.8664 (5)	0.6385 (6)	0.5193 (4)	0.108 (2)	
H12	0.8997	0.6032	0.5582	0.129*	
C13	0.7513 (4)	0.6482 (4)	0.5093 (2)	0.0674 (13)	
H13	0.7081	0.6214	0.5421	0.081*	
C14	0.5351 (3)	0.6152 (3)	0.69330 (16)	0.0374 (8)	
C15	0.5009 (4)	0.7003 (4)	0.73770 (18)	0.0523 (10)	
H15	0.4260	0.7099	0.7425	0.063*	
C16	0.5793 (4)	0.7712 (4)	0.7751 (2)	0.0650 (12)	
H16	0.5563	0.8270	0.8054	0.078*	
C17	0.6881 (4)	0.7596 (4)	0.7676 (2)	0.0667 (13)	
H17	0.7393	0.8089	0.7919	0.080*	
C18	0.7238 (4)	0.6755 (4)	0.7245 (2)	0.0620 (11)	
H18	0.7990	0.6668	0.7202	0.074*	
C19	0.6475 (3)	0.6034 (3)	0.6872 (2)	0.0479 (9)	
H19	0.6719	0.5467	0.6579	0.057*	
C20	0.3075 (3)	0.5396 (3)	0.67562 (18)	0.0447 (9)	
C21	0.2194 (4)	0.6045 (4)	0.6469 (2)	0.0674 (13)	
H21	0.2235	0.6428	0.6060	0.081*	
C22	0.1227 (4)	0.6127 (6)	0.6800 (3)	0.101 (2)	
H22	0.0635	0.6591	0.6618	0.121*	
C23	0.1159 (5)	0.5525 (7)	0.7387 (3)	0.097 (2)	
H23	0.0509	0.5561	0.7596	0.117*	
C24	0.2022 (5)	0.4879 (6)	0.7670 (3)	0.0867 (17)	
H24	0.1966	0.4478	0.8072	0.104*	
C25	0.2989 (4)	0.4813 (4)	0.7361 (2)	0.0630 (12)	
H25	0.3586	0.4376	0.7559	0.076*	
C26	0.9176 (7)	0.9290 (8)	0.6327 (6)	0.124 (3)	
C27	0.8934 (8)	1.0016 (10)	0.5727 (5)	0.182 (4)	
H27A	0.8406	0.9604	0.5420	0.273*	
H27B	0.9603	1.0147	0.5517	0.273*	
H27C	0.8633	1.0775	0.5846	0.273*	

### Atomic displacement parameters (Å^2^)

**Table:**

	*U*^11^	*U*^22^	*U*^33^	*U*^12^	*U*^13^	*U*^23^
Ag1	0.04039 (16)	0.03674 (17)	0.03093 (15)	0.00023 (11)	0.00592 (11)	0.00355 (11)
P1	0.0365 (5)	0.0316 (5)	0.0294 (4)	−0.0030 (4)	0.0052 (4)	0.0017 (4)
P2	0.0383 (5)	0.0328 (5)	0.0274 (4)	−0.0006 (4)	0.0060 (4)	0.0002 (4)
N1	0.045 (2)	0.081 (3)	0.060 (2)	0.0081 (19)	−0.0008 (17)	0.015 (2)
N2	0.131 (6)	0.147 (7)	0.176 (8)	0.016 (5)	−0.029 (6)	−0.015 (6)
O1	0.0686 (18)	0.088 (2)	0.0610 (19)	0.0234 (16)	0.0020 (16)	−0.0021 (15)
O2	0.0537 (17)	0.069 (2)	0.0640 (18)	0.0051 (15)	−0.0012 (12)	−0.0018 (13)
C1	0.0462 (19)	0.0323 (18)	0.0326 (18)	−0.0010 (15)	0.0057 (15)	0.0028 (14)
C2	0.051 (2)	0.0306 (18)	0.0323 (18)	−0.0031 (15)	0.0126 (16)	0.0013 (14)
C3	0.058 (2)	0.035 (2)	0.056 (2)	−0.0002 (18)	0.0018 (19)	0.0038 (17)
C4	0.080 (3)	0.050 (3)	0.065 (3)	0.014 (2)	0.008 (2)	0.015 (2)
C5	0.111 (4)	0.039 (2)	0.060 (3)	0.009 (3)	0.034 (3)	0.013 (2)
C6	0.096 (4)	0.033 (2)	0.061 (3)	−0.015 (2)	0.031 (3)	−0.009 (2)
C7	0.061 (2)	0.044 (2)	0.043 (2)	−0.0107 (19)	0.0157 (18)	−0.0063 (17)
C8	0.0367 (18)	0.040 (2)	0.047 (2)	−0.0064 (15)	0.0066 (16)	−0.0043 (17)
C9	0.051 (2)	0.087 (3)	0.059 (3)	−0.016 (2)	0.020 (2)	−0.007 (2)
C10	0.057 (3)	0.115 (5)	0.112 (5)	−0.020 (3)	0.039 (3)	−0.021 (4)
C11	0.034 (3)	0.118 (5)	0.176 (7)	−0.006 (3)	0.009 (4)	−0.010 (5)
C12	0.053 (3)	0.124 (5)	0.139 (6)	0.000 (3)	−0.029 (4)	0.038 (4)
C13	0.049 (2)	0.075 (3)	0.076 (3)	−0.004 (2)	−0.005 (2)	0.021 (3)
C14	0.050 (2)	0.0329 (18)	0.0287 (17)	−0.0038 (15)	0.0016 (15)	0.0020 (14)
C15	0.067 (3)	0.052 (2)	0.040 (2)	−0.004 (2)	0.0121 (19)	−0.0085 (18)
C16	0.096 (4)	0.055 (3)	0.043 (2)	−0.011 (3)	0.006 (2)	−0.017 (2)
C17	0.082 (3)	0.058 (3)	0.055 (3)	−0.017 (2)	−0.021 (2)	−0.006 (2)
C18	0.054 (2)	0.065 (3)	0.064 (3)	−0.006 (2)	−0.011 (2)	0.000 (2)
C19	0.048 (2)	0.046 (2)	0.048 (2)	0.0000 (17)	−0.0022 (18)	−0.0030 (18)
C20	0.048 (2)	0.050 (2)	0.039 (2)	−0.0062 (18)	0.0155 (17)	−0.0082 (17)
C21	0.051 (2)	0.095 (4)	0.057 (3)	0.011 (2)	0.014 (2)	0.001 (3)
C22	0.050 (3)	0.152 (6)	0.104 (5)	0.020 (3)	0.023 (3)	−0.006 (4)
C23	0.073 (4)	0.136 (6)	0.090 (4)	−0.018 (4)	0.049 (3)	−0.028 (4)
C24	0.091 (4)	0.106 (4)	0.071 (3)	−0.021 (4)	0.049 (3)	−0.008 (3)
C25	0.070 (3)	0.072 (3)	0.051 (3)	−0.008 (2)	0.024 (2)	0.006 (2)
C26	0.106 (6)	0.106 (6)	0.158 (9)	0.002 (5)	−0.001 (6)	−0.026 (6)
C27	0.182 (9)	0.176 (10)	0.190 (10)	−0.011 (8)	0.035 (8)	0.040 (9)

### Geometric parameters (Å, º)

**Table:**

Ag1—P2	2.4396 (9)	C10—H10	0.9300
Ag1—P1	2.4747 (9)	C11—C12	1.360 (9)
Ag1—O1	2.550 (3)	C11—H11	0.9300
Ag1—O2	2.567 (3)	C12—C13	1.396 (7)
Ag1—Ag1^i^	3.1809 (5)	C12—H12	0.9300
P1—C8	1.816 (3)	C13—H13	0.9300
P1—C2	1.819 (3)	C14—C19	1.388 (5)
P1—C1	1.839 (3)	C14—C15	1.390 (5)
P2—C20	1.824 (4)	C15—C16	1.397 (6)
P2—C14	1.827 (3)	C15—H15	0.9300
P2—C1^i^	1.839 (3)	C16—C17	1.350 (6)
N1—O2	1.229 (4)	C16—H16	0.9300
N1—O1	1.248 (5)	C17—C18	1.372 (6)
N2—C26	1.144 (11)	C17—H17	0.9300
C1—P2^i^	1.839 (3)	C18—C19	1.387 (6)
C1—H1A	0.9700	C18—H18	0.9300
C1—H1B	0.9700	C19—H19	0.9300
C2—C3	1.388 (5)	C20—C21	1.371 (6)
C2—C7	1.389 (5)	C20—C25	1.387 (5)
C3—C4	1.382 (5)	C21—C22	1.405 (6)
C3—H3	0.9300	C21—H21	0.9300
C4—C5	1.365 (7)	C22—C23	1.364 (9)
C4—H4	0.9300	C22—H22	0.9300
C5—C6	1.363 (7)	C23—C24	1.349 (8)
C5—H5	0.9300	C23—H23	0.9300
C6—C7	1.387 (6)	C24—C25	1.381 (6)
C6—H6	0.9300	C24—H24	0.9300
C7—H7	0.9300	C25—H25	0.9300
C8—C13	1.369 (6)	C26—C27	1.455 (12)
C8—C9	1.387 (5)	C27—H27A	0.9600
C9—C10	1.376 (7)	C27—H27B	0.9600
C9—H9	0.9300	C27—H27C	0.9600
C10—C11	1.353 (9)		
			
P2—Ag1—P1	144.82 (3)	C11—C10—C9	121.2 (5)
P2—Ag1—O1	106.05 (8)	C11—C10—H10	119.4
P1—Ag1—O1	102.83 (8)	C9—C10—H10	119.4
P2—Ag1—O2	129.40 (7)	C10—C11—C12	120.1 (5)
P1—Ag1—O2	85.19 (7)	C10—C11—H11	119.9
O1—Ag1—O2	47.95 (10)	C12—C11—H11	119.9
P2—Ag1—Ag1^i^	90.06 (2)	C11—C12—C13	119.8 (6)
P1—Ag1—Ag1^i^	78.39 (2)	C11—C12—H12	120.1
O1—Ag1—Ag1^i^	143.11 (8)	C13—C12—H12	120.1
O2—Ag1—Ag1^i^	96.21 (7)	C8—C13—C12	120.2 (5)
C8—P1—C2	104.87 (17)	C8—C13—H13	119.9
C8—P1—C1	104.07 (17)	C12—C13—H13	119.9
C2—P1—C1	102.52 (15)	C19—C14—C15	118.5 (3)
C8—P1—Ag1	121.84 (12)	C19—C14—P2	119.5 (3)
C2—P1—Ag1	113.54 (11)	C15—C14—P2	121.4 (3)
C1—P1—Ag1	107.99 (11)	C14—C15—C16	119.8 (4)
C20—P2—C14	103.94 (16)	C14—C15—H15	120.1
C20—P2—C1^i^	105.18 (17)	C16—C15—H15	120.1
C14—P2—C1^i^	103.96 (16)	C17—C16—C15	120.7 (4)
C20—P2—Ag1	118.75 (13)	C17—C16—H16	119.7
C14—P2—Ag1	110.93 (11)	C15—C16—H16	119.7
C1^i^—P2—Ag1	112.72 (11)	C16—C17—C18	120.4 (4)
O2—N1—O1	114.1 (3)	C16—C17—H17	119.8
N1—O1—Ag1	99.1 (2)	C18—C17—H17	119.8
N1—O2—Ag1	98.8 (2)	C17—C18—C19	119.9 (4)
P1—C1—P2^i^	110.85 (18)	C17—C18—H18	120.1
P1—C1—H1A	109.5	C19—C18—H18	120.1
P2^i^—C1—H1A	109.5	C18—C19—C14	120.6 (4)
P1—C1—H1B	109.5	C18—C19—H19	119.7
P2^i^—C1—H1B	109.5	C14—C19—H19	119.7
H1A—C1—H1B	108.1	C21—C20—C25	119.4 (4)
C3—C2—C7	117.9 (3)	C21—C20—P2	119.4 (3)
C3—C2—P1	119.7 (3)	C25—C20—P2	121.1 (3)
C7—C2—P1	122.3 (3)	C20—C21—C22	119.3 (5)
C4—C3—C2	120.9 (4)	C20—C21—H21	120.3
C4—C3—H3	119.6	C22—C21—H21	120.3
C2—C3—H3	119.6	C23—C22—C21	119.9 (6)
C5—C4—C3	120.2 (4)	C23—C22—H22	120.1
C5—C4—H4	119.9	C21—C22—H22	120.1
C3—C4—H4	119.9	C24—C23—C22	121.0 (5)
C6—C5—C4	120.2 (4)	C24—C23—H23	119.5
C6—C5—H5	119.9	C22—C23—H23	119.5
C4—C5—H5	119.9	C23—C24—C25	120.0 (5)
C5—C6—C7	120.2 (4)	C23—C24—H24	120.0
C5—C6—H6	119.9	C25—C24—H24	120.0
C7—C6—H6	119.9	C24—C25—C20	120.4 (5)
C6—C7—C2	120.6 (4)	C24—C25—H25	119.8
C6—C7—H7	119.7	C20—C25—H25	119.8
C2—C7—H7	119.7	N2—C26—C27	178.9 (11)
C13—C8—C9	119.1 (4)	C26—C27—H27A	109.5
C13—C8—P1	118.6 (3)	C26—C27—H27B	109.5
C9—C8—P1	122.2 (3)	H27A—C27—H27B	109.5
C10—C9—C8	119.5 (5)	C26—C27—H27C	109.5
C10—C9—H9	120.2	H27A—C27—H27C	109.5
C8—C9—H9	120.2	H27B—C27—H27C	109.5
			
P2—Ag1—P1—C8	11.22 (16)	C4—C5—C6—C7	0.3 (7)
O1—Ag1—P1—C8	155.83 (16)	C5—C6—C7—C2	−1.1 (6)
O2—Ag1—P1—C8	−159.29 (16)	C3—C2—C7—C6	0.8 (5)
Ag1^i^—Ag1—P1—C8	−61.93 (14)	P1—C2—C7—C6	−175.4 (3)
P2—Ag1—P1—C2	−115.66 (13)	C2—P1—C8—C13	128.7 (3)
O1—Ag1—P1—C2	28.95 (15)	C1—P1—C8—C13	−124.0 (3)
O2—Ag1—P1—C2	73.83 (14)	Ag1—P1—C8—C13	−1.9 (4)
Ag1^i^—Ag1—P1—C2	171.19 (13)	C2—P1—C8—C9	−52.9 (4)
P2—Ag1—P1—C1	131.39 (12)	C1—P1—C8—C9	54.4 (4)
O1—Ag1—P1—C1	−84.01 (14)	Ag1—P1—C8—C9	176.5 (3)
O2—Ag1—P1—C1	−39.13 (13)	C13—C8—C9—C10	−0.1 (7)
Ag1^i^—Ag1—P1—C1	58.23 (12)	P1—C8—C9—C10	−178.5 (4)
P1—Ag1—P2—C20	156.55 (14)	C8—C9—C10—C11	1.1 (9)
O1—Ag1—P2—C20	12.54 (16)	C9—C10—C11—C12	−0.6 (11)
O2—Ag1—P2—C20	−35.73 (17)	C10—C11—C12—C13	−0.8 (11)
Ag1^i^—Ag1—P2—C20	−133.82 (14)	C9—C8—C13—C12	−1.3 (7)
P1—Ag1—P2—C14	36.27 (14)	P1—C8—C13—C12	177.1 (4)
O1—Ag1—P2—C14	−107.73 (15)	C11—C12—C13—C8	1.8 (9)
O2—Ag1—P2—C14	−156.00 (15)	C20—P2—C14—C19	163.7 (3)
Ag1^i^—Ag1—P2—C14	105.91 (12)	C1^i^—P2—C14—C19	53.8 (3)
P1—Ag1—P2—C1^i^	−79.84 (14)	Ag1—P2—C14—C19	−67.6 (3)
O1—Ag1—P2—C1^i^	136.15 (15)	C20—P2—C14—C15	−25.3 (3)
O2—Ag1—P2—C1^i^	87.89 (16)	C1^i^—P2—C14—C15	−135.1 (3)
Ag1^i^—Ag1—P2—C1^i^	−10.21 (13)	Ag1—P2—C14—C15	103.5 (3)
O2—N1—O1—Ag1	−2.0 (4)	C19—C14—C15—C16	−0.2 (5)
P2—Ag1—O1—N1	−127.9 (2)	P2—C14—C15—C16	−171.3 (3)
P1—Ag1—O1—N1	72.4 (3)	C14—C15—C16—C17	1.2 (6)
O2—Ag1—O1—N1	1.2 (2)	C15—C16—C17—C18	−1.8 (7)
Ag1^i^—Ag1—O1—N1	−15.2 (3)	C16—C17—C18—C19	1.3 (7)
O1—N1—O2—Ag1	2.0 (4)	C17—C18—C19—C14	−0.2 (6)
P2—Ag1—O2—N1	73.8 (3)	C15—C14—C19—C18	−0.3 (6)
P1—Ag1—O2—N1	−113.3 (2)	P2—C14—C19—C18	171.0 (3)
O1—Ag1—O2—N1	−1.2 (2)	C14—P2—C20—C21	113.3 (3)
Ag1^i^—Ag1—O2—N1	169.0 (2)	C1^i^—P2—C20—C21	−137.8 (3)
C8—P1—C1—P2^i^	62.7 (2)	Ag1—P2—C20—C21	−10.5 (4)
C2—P1—C1—P2^i^	171.77 (18)	C14—P2—C20—C25	−64.7 (4)
Ag1—P1—C1—P2^i^	−68.08 (18)	C1^i^—P2—C20—C25	44.2 (4)
C8—P1—C2—C3	157.2 (3)	Ag1—P2—C20—C25	171.5 (3)
C1—P1—C2—C3	48.7 (3)	C25—C20—C21—C22	1.2 (7)
Ag1—P1—C2—C3	−67.5 (3)	P2—C20—C21—C22	−176.8 (4)
C8—P1—C2—C7	−26.7 (3)	C20—C21—C22—C23	−2.5 (9)
C1—P1—C2—C7	−135.1 (3)	C21—C22—C23—C24	2.1 (10)
Ag1—P1—C2—C7	108.7 (3)	C22—C23—C24—C25	−0.5 (9)
C7—C2—C3—C4	0.3 (6)	C23—C24—C25—C20	−0.8 (8)
P1—C2—C3—C4	176.6 (3)	C21—C20—C25—C24	0.4 (7)
C2—C3—C4—C5	−1.1 (6)	P2—C20—C25—C24	178.4 (4)
C3—C4—C5—C6	0.8 (7)		

Symmetry code: (i) −*x*+1, −*y*+1, −*z*+1.
